# Toxic Effects of Different Coating-Related Functionalized Nanoparticles on Aquatic Organisms

**DOI:** 10.3390/toxics12020142

**Published:** 2024-02-09

**Authors:** David Hernández-Moreno, Marta Fernández-Díaz, Isabel Rucandio, José María Navas, María Luisa Fernández-Cruz

**Affiliations:** 1 Department of Environment and Agronomy, National Institute of Agriculture and Food Research and Technology (INIA), Spanish National Research Council (CSIC), Carretera de A Coruña Km 7, 28040 Madrid, Spain; jmnavas@inia.csic.es; 2 Research Centre for Energy, Environment and Technology (CIEMAT), Av. Complutense, 40, 28040 Madrid, Spain; marta.fernandez@ciemat.es (M.F.-D.); isabel.rucandio@ciemat.es (I.R.)

**Keywords:** nanoform, in vitro, *Daphnia magna*, rainbow trout, surface chemistry

## Abstract

The peculiar physico-chemical characteristics of nanomaterials (NMs) and the use of different coatings to improve their expected properties result in a huge amount of nanoforms, which vary in chemical composition, size, shape and surface characteristics. This makes it almost impossible to test all the nanoforms available, and efforts have been made to establish grouping or read-across strategies. The aim of this work was to find a behavior pattern of effect among nanoforms of different metallic core nanoparticles (NPs) (TiO_2_, CeO_2_ and Ag NP) with the same coatings (sodium citrate, poly (ethylene glycol), dodecylphosphonic acid or oleylamine). *Daphnia magna*, rainbow trout and two fish cell lines (PLHC-1 and RTH-149) were exposed to a range of concentrations (up to 100 mg/L) of the uncoated or coated NPs. Ag NPs were the most toxic, followed by CeO_2_ NPs and finally by TiO_2_ NPs. The results show that a clear pattern of toxicity in the studied species could not be established related to the coatings. However, it was possible to confirm different inter-species sensitivities. RTH-149 was the most sensitive cell line, and *Daphnia magna* was more sensitive than fish. Moreover, some differences in coating-core interactions were found between the metal oxide and the metal NPs in *Daphnia magna*.

## 1. Introduction

Manufactured nanomaterials (NMs) include those nanoparticles (NPs) synthesized and modified in order to enhance their performance in several technological and industrialized processes. For this reason, there is a wide variety of NMs on the market with different core composition, sizes, shapes and surface properties. The advances in nanotechnology have favored an increase in the use of coatings to modify the core NM surface. These coatings are used as part of the safe-by-design strategy to improve, for instance, the reactivity of NMs with a target organism or their dispersion and stability or to reduce their hazard or fate persistence [1,2].

The physico-chemical properties of the NMs are key elements in determining their fate and behavior in aquatic systems [3,4,5]. For this reason, it is of high importance to systematically and accurately characterize the NMs used in (eco)toxicity studies [6] and to provide information about the properties that determine their biological effects [7]. In (eco)toxicity studies, size, shape and surface charge (zeta (ζ) potential) have been mentioned as the most relevant properties that should be characterized. These properties need to be evaluated for pristine NMs (dry powdered or liquid stock) and for NMs in the exposure media. In addition, any other relevant characteristic of the NM can improve the interpretation of the relationship between the physico-chemical property and the effect observed [6,8,9]. Knowledge on how these properties influence toxicity would help in the development of read-across approaches for NMs. Over the last decade, there have been several projects (MARINA, NanoMILE, NANoREG, GUIDEnano, NanoREG II, GRACIOUS) working on approaches such as grouping, equivalence and read-across based on NMs’ physico-chemical properties. This task is of high priority in regulatory frameworks, even at the Organization for Economic Co-operation and Development (OECD) where several approaches related to these issues are being developed [10].

Our main goal was to evaluate how the functionalization acquired through the addition of hydrophilic or hydrophobic coatings modulates the toxic effect of two metal oxide NPs (titanium dioxide (TiO_2_ NPs) and cerium dioxide (CeO_2_ NPs)) and one metal NP (silver (Ag NPs)) in different aquatic species. We followed in vitro and in vivo approaches. To perform the cytotoxicity assays, two liver cell lines from two fish species *Poeciliopsis lucida* (clearfin livebearer) (PLHC-1) and *Oncorhynchus mykiss* (rainbow trout) (RTH-149) were chosen since liver has been reported as the major site of clearance after an exposure of fish to chemicals. In addition, these fish species grow in warm and cold temperatures, respectively. Acute toxicity assays were also conducted in two aquatic species representative of different taxa, the invertebrate *Daphnia magna* and the vertebrate rainbow trout. The results of this study may contribute to understanding the behavior of NPs depending on their coating and could be used to develop rules intended for the grouping of NMs. In addition, the comparison between in vitro/in vivo findings would be of interest for further validations of in vitro assay results.

## 2. Materials and Methods

### 2.1. Nanomaterials and Chemicals

All the uncoated and coated NPs used in this study were provided by PlasmaChem GmbH (Berlin, Germany) as suspensions (Table S1) (Berlin, Germany). Two metal oxide NPs (TiO_2_ and CeO_2_, 4–8 nm) and one metal NP (Ag NP, 10 nm) were covered with two hydrophilic coatings, sodium citrate (CIT) and poly (ethylene glycol) (PEG), and two hydrophobic ones, dodecylphosphonic acid (DDPA) and oleylamine (OAM). These coatings are very often used to provide these different hydrophilicities. The Ag-CIT NPs were considered the uncoated Ag NPs, since due to its weak bound to the core Ag [11], this coating is easily lost once the NPs are suspended in an aqueous medium. Therefore, the obtained manufactured NPs were TiO_2_ uncoated (TiO_2_-UNC), TiO_2_-CIT, TiO_2_-PEG, TiO_2_-DDPA, CeO_2_-UNC, CeO_2_-CIT, CeO_2_-PEG, CeO_2_-DDPA, Ag-CIT, Ag-PEG and Ag-OAM. Ag NPs could not be coated with DDPA, since it led to insufficient coating and resulted in a strong coagulation of the particles in hydrophobic solvents. The same problem occurred when OAM was used with TiO_2_ and CeO_2_ NPs. Ag NPs were stabilized with OAM, which complexed strongly with the surface of this metal by its NH_2_ group. On the other hand, DDPA was covalently bound successfully to the surface of the TiO_2_ and CeO_2_ NPs. PlasmaChem GmbH also provided the vehicles used for each NP, containing the solvent and the remaining coating substance (not bound) after removing the NP fraction via filtration. They also supplied solutions with the complete amount of coating (bound + not bound) and without the core NPs. L-glutamine (200 mM), fetal bovine serum (FBS), penicillin and streptomycin (P/S) (10,000 U/mL/10 mg/mL) and cell culture EMEM (Eagle’s Minimum Essential Medium) medium (ref.: BE12-662F and BE12-125F) were purchased from Lonza (Barcelona, Spain). AlamarBlue (AB) reagent was purchased from Invitrogen (Thermo Fisher Scientific; Madrid, Spain). Neutral red (NR) solution (0.33%), 5-carboxyfluorescein diacetate-acetoxymethyl ester (CFDA-AM), sodium dodecyl sulphate and glacial acetic acid were from Sigma-Aldrich (Madrid, Spain). Ethanol was from Panreac (Barcelona, Spain). All solutions used for inductively coupled plasma optical emission spectrometry (ICP-OES) and inductively coupled plasma mass spectrometry (ICP-MS) analyses were prepared with analytical reagent-grade chemicals from Merck KGaA (Darmstadt, Germany) and ultrapure water (18 MΩ cm). Standard certified mono-elemental solutions containing 1000 mg/L of Ti, Ce and Ag and standard solutions containing 1000 mg/L of gallium (Ga), indium (In) and lutetium (Lu) were purchased from Analytika (Spol S.R.O., Staré Město, Czech Republic) and were used to prepare the calibration and internal standards for ICP-MS, respectively. Argon gas of a purity higher than 99.999% was used for the ICP-OES and ICP-MS instruments (Air Liquide, Madrid, Spain). The acute toxicity assays in *Daphnia magna* were developed using the Daphtoxkit (MicroBioTests, Ghent, Belgium) distributed by Ecotest (Valencia, Spain).

### 2.2. Characterization of the NPs

The sizes, surface charges, shapes and concentrations of the NPs were measured in the stock suspensions and in the different exposure media at the maximum exposure concentrations. Transmission electron microscopy (TEM), dynamic light scattering (DLS), ICP-OES and ICP-MS techniques were used.

#### 2.2.1. TEM Analyses

The sizes and the shapes of the NPs in the stock suspensions and after dispersion in the exposure media at the maximum exposure concentrations (time 0) were characterized via TEM. Samples were prepared by dropping aliquots of the NP suspensions onto carbon-coated grids and allowing them to dry for some minutes. The microscope used was a JEOL JEM-2100 HT (JEOL Ltd., Tokyo, Japan) operated at an accelerating voltage of 200 kV with integrated energy dispersive X-ray (EDX) spectroscopy (Oxford Inca, Oxford, UK). The analyses were performed at the Centro Nacional de Microscopía Electrónica (ICTS-CNME, Madrid, Spain). The sizes of the particles (ferret diameter) in the TEM micrographs were measured using the image processing and analysis software ImageJ version 1.34u (Wayne Rasband, National Institutes of Health, New York, NY, USA).

#### 2.2.2. DLS Measurements

The hydrodynamic sizes of the particles in suspension were determined using a Zetasizer Nano-ZS (Malvern Instruments Ltd., Worcestershire, UK). Measurements were performed in the exposure suspensions at the maximum concentrations directly after preparation and at the end of the assays, 24 h for cytotoxicity assays in fish cell lines and 48 h for the *Daphnia magna* test. The hydrodynamic sizes of the NPs in the fish exposure tanks were also measured daily during the 96 h acute toxicity assay by taking aliquots from the water column. Medium without NPs was used as a control and to record any background signals that may arise from medium components. Before preparing the samples, the instrument temperature was set to the corresponding exposure condition temperature. Four independent measurements were taken with each measurement consisting of six runs of 20 s durations. 

The ζ-potential was measured for the uncoated and coated (CIT and PEG) metal oxide NPs at the highest exposure concentration once dispersed in the media. Due to limitations in the stock concentration, this property was not measured for Ag NPs. ζ-potential measurements were performed using disposable capillary cuvettes (Malvern Instruments Ltd.). Three measurements were taken of each sample and the number of runs was set automatically.

#### 2.2.3. Metal Analyses via ICP-MS and ICP-OES

The stock suspensions of the coated and uncoated TiO_2_, CeO_2_ and Ag NPs, as well as their vehicles and their suspensions in the exposure media at the maximum concentrations assayed, were analyzed to determine the Ti, Ce and Ag contents. ICP-OES was used for concentrations higher than 0.05 mg/L and ICP-MS for lower concentrations. During the assays with rainbow trout, water samples were taken daily from time 0 to time 96 h to measure the metal concentrations. ICP-OES analyses were conducted by using a benchtop dual view ICP-OES with a vertical torch, Agilent 5100 model (Agilent Technologies Spain, S.L., Madrid, Spain). Two or three different wavelengths were used for the Ce, Ti and Ag quantifications (399.934, 407.570, and 418.659 nm for Ce, 334.941, 336.122 and 337.280 nm for Ti and 328.068 and 338.289 nm for Ag), and the averages of the three lines were considered. Samples were shaken immediately before measuring. ICP-MS analyses were conducted with a Thermo iCAP-Q (Thermo Scientific, Darmstadt, Germany) spectrometer equipped with a quadrupole mass analyzer and an electron multiplier detector. A Meinhard nebulizer (Meinhard, Golden, CO, USA) with a baffled cyclonic spray chamber and a peristaltic pump were used for sample introduction. All analyses were performed in collision cell mode with helium gas and kinetic energy discrimination, to overcome isobaric and polyatomic interferences, mainly in the Ti isotopes. Limits of detection (LOD) and limits of quantification (LOQ) were calculated as being 3 or 10 times the standard deviation of the blank, respectively. 

For Ti and Ce content determinations, the samples were prepared via closed-vessel microwave digestion in an Ethos 1 microwave (Milestone, Sorisole, Italy) with a mixture of nitric acid (HNO_3_), hydrogen peroxide (H_2_O_2_) and hydrofluoric acid (HF). After cooling at room temperature, the digested samples were transferred into polyethylene flasks and made up to volume with ultrapure water before analysis. The samples with Ag NPs were also mineralized via microwave with HNO_3_ and H_2_O_2_, and directly measured via ICP-OES or ICP-MS. For those samples where any Cl^−^ could be present, such as in the cell culture media, the digested suspensions were centrifuged (1660× *g*, 15 min) to separate any AgCl precipitate. The supernatants were collected and diluted with ammonium hydroxide (NH_4_OH, 2.8% *w*/*w*), while the precipitate, if it appeared, was dissolved with concentrated NH_4_OH, and then diluted with NH_4_OH (2.8% *w*/*w*) and stored for subsequent analysis.

The amounts measured for Ti and Ce are reported in the text as TiO_2_ and CeO_2_. 

### 2.3. Cell Culture and Exposure

Two different hepatocellular fish cells were used to perform the cytotoxicity experiments. The hepatocellular carcinoma cell line PLHC-1, derived from topminnow fish (*Poeciliopsis lucida*), and the hepatoma cell line RTH-149, derived from rainbow trout (*Oncorhynchus mykiss*), were obtained from the American Type Culture Collection (ATCC, Manassas, VA, USA). PLHC-1 cells were cultured at 30 °C and 5% CO_2_ in EMEM ref. BE12-662F supplemented with 5% fetal bovine serum (FBS), 1% P/S and 1% L-glutamine. RTH-149 cells were cultured at 20 °C and 5% CO_2_ in EMEM ref. BE12-125F supplemented with 10% FBS, 1% P/S, 1% L-glutamine and 1% sodium pyruvate. Cells (100 µL suspension) were seeded in 96 well plates (PLHC-1 at 50 × 10^3^ cells per well and RTH-149 at 28 × 10^3^ cells per well) and maintained under their specific environmental conditions for 24 h to obtain a confluent cell monolayer. Both cell lines were exposed to NPs in the 96 well plates (Greiner Bio-one GmbH; Madrid, Spain). The working suspensions of each NP were prepared by directly pipetting the stock suspension into the corresponding medium and then vortexed for 1 min just before applying them to the cells. The final nominal concentrations of TiO_2_ NPs and CeO_2_ NPs calculated in relation to the core NPs ranged from 0.78 to 100 mg TiO_2_ or CeO_2_/L. This range was selected by taking into account the recommended maximum concentrations according to OECD guidelines for in vivo experiments with aquatic organisms (100 mg/L). However, lower ranges of nominal concentrations were applied for Ag-CIT (0.078 to 10 mg Ag/L), Ag-PEG (0.013 to 1.62 mg Ag/L) and Ag-OAM (0.068 to 8.66 mg Ag/L) due to the high toxicity reported for Ag NPs (IC_50s_ of 10.7–19.8 mg/L) to rainbow trout cell lines [12] and due to limitations in the stock concentrations. Cells were also treated with concentrated coatings (Table S1) to verify their own toxicity. Dilutions of these suspensions were prepared in the same way as the NP working concentrations (8 concentrations at ½ dilution ratio). A vehicle, positive and negative controls were included in each plate. The vehicle control consisted of the vehicle supplied by PlasmaChem for each NP, diluted in the cell culture medium at the percentage the vehicle is present at the highest tested concentration of the NP (0.07–3%). The positive control used was sodium dodecyl sulphate at a 66–500 μM range. The negative control was cells in their medium. Exposure lasted for 24 h, after which the medium was removed, and three cytotoxicity assays were performed. 

#### Cytotoxicity Assays

The fluorometric-based assay system described by Dayeh et al. [13], with the modifications reported by Lammel et al. [14], was followed. This protocol facilitates the simultaneous use of three assays to monitor different endpoints of cytotoxicity, following 24 h of exposure to NPs. This system allows the mechanism of action by which NPs can interfere with cellular homeostasis to be determined. The Alamar Blue (AB) test evaluates cell viability based on mitochondrial activity; the 5-carboxyfluorescein diacetate, acetoxymethyl ester (CFDA-AM) assay indicates damage to the plasma membrane; and the neutral red uptake (NRU) assay determines the accumulation of neutral red dye in the lysosomes of viable, uninjured cells. The absence of interferences of the NPs was checked before developing the cytotoxicity assays, following the method reported by Connolly et al. [12].

### 2.4. In Vivo Acute Toxicity Assays

#### 2.4.1. Toxicity Assays in Daphnia Magna

Immobilization tests were performed in accordance with the OECD test guideline (TG) no. 202 [15]. The tests were performed using the Daphtoxkit. Firstly, a limit test assay was developed, followed by a dose–response assay with those NPs showing toxicity in the limit test. Three days prior to the start of the toxicity test, the hatching of the ephippias was initiated, via incubation in pre-aerated standard freshwater (72 h, 21 °C and continuous illumination of ~6000 lux). Neonates were fed for 2 h prior to the test with *Spirulina microalgae* (spirulina). Five neonates (<24 h old), randomly chosen, were placed in 10 mL test wells per replicate (four replicates) and exposed to a range of concentrations (120 animals in total per substance assayed). The nominal concentrations ranged from 0.01 to 100 mg TiO_2_ or CeO_2_/L for coated and uncoated TiO_2_ and CeO_2_ NPs. Due to limitations in the amount of stock of Ag NPs, the nominal concentrations of Ag NPs were much lower (0.0001 to 1 mg Ag/L for Ag-CIT, 0.000016 to 0.0162 mg Ag/L for Ag-PEG and 0.000087 to 0.866 mg Ag/L for Ag-OAM). Tests were conducted at a constant temperature of 21 °C in the dark and daphnids were not fed during the experiments. After 24 h and 48 h, immobilization was recorded, and the effective concentration (EC_50_) values were calculated. 

#### 2.4.2. Toxicity Assays in Fish

Acute toxicity tests following the OECD TG no. 203 [16] were performed in juveniles of rainbow trout. Fish with a mean total body weight of 3.14 ± 0.76 g (mean ± SD) and length of 6.15 ± 0.50 cm were obtained from a trout farm (Fish Farm of Escuela Técnica Superior Ingenieros de Montes, Universidad Politécnica, Madrid, Spain). The fish were kept at INIA fish facilities in 600 L tanks. After 15 days of acclimation, 7 fish were transferred to rectangular 33 L tanks supplied with flow-through water taken from a tank with filtered reconstituted water (according to OECD parameters) and allowed to adapt for 10 days prior to starting the experiments. During this period, the fish were maintained under controlled conditions and fed with a commercial diet for trout, at a rate of 2% of their body weight. During the whole experiment, the photoperiod was 12/12, the temperature oscillated between 12.4 °C and 14.5 °C, the dissolved oxygen remained always higher than 75%, and the pH remained between 7.4 and 7.8. No mortality was observed during the acclimation period, or for the controls during the whole experiment. Limit tests were carried out, exposing fish for 96 h, under static conditions, to 100 mg TiO_2_ or CeO_2_/L of either TiO_2_ NPs or CeO_2_ NPs (uncoated or coated with –CIT or –PEG), as well as to the vehicles. Ninety fish were used in total and distributed in 2 aquaria for control fish, 6 aquaria for the six different vehicles and 6 aquaria for the exposure to the six NPs. Due to the hydrophobic character of DDPA, a good and stable dispersion was not reached under the conditions of preparation of the exposure media. For this reason, the assay was not performed with these NPs. Ag NPs were tested in a range of 5 nominal concentrations up to maximums of 1.5 mg Ag/L (Ag-CIT) and 0.24 mg Ag/L (Ag-PEG) due to limitations in the amount of stock NPs. Ninety fish were used in total and distributed in 2 aquaria for control fish, 2 aquaria for the two different vehicles and 10 aquaria for the exposure to the five different concentrations of each one of the Ag NPs. Fish were observed at 0, 2, 4, 6, 24, 48, 72 and 96 h. All the experiments were performed according to the EU and national legislation for the use of laboratory animals for scientific purposes after receiving a favorable report from the INIA ethical committee for laboratory experimentation and the corresponding authorization from the competent authority at the Community of Madrid regional government (PROEX 37/13). 

### 2.5. Statistical Analysis

The raw data of the cytotoxicity assays were corrected by subtracting the background fluorescence (cell-free control) and normalized as percentages against the vehicle control. All results were presented as mean ± standard error of the mean (SEM) of at least three independent experiments performed in triplicate. The results obtained for a given concentration in the coated NP experiments were compared with the corresponding results of cells exposed to the uncoated metal NP suspensions. Statistical analyses were also performed for individual treatments of the same NP, comparing the result obtained for each concentration with the control cells. Raw data from *Daphnia magna* assays were transformed into percentages of survival, making comparisons between each exposed group and the control. The normality and homoscedasticity of all data were checked using the Shapiro–Wilk test and Bartlett’s test, respectively. A parametric one-way analysis of variance (ANOVA) followed by a Dunnett’s post hoc test was applied for all these statistical analyses. The estimation of the concentration–response function and the calculation of the I(E)C_20/50_ (concentration causing a 20% or 50% of inhibition/effect with respect to the controls) were performed by fitting the assay results to a regression model equation for a sigmoidal curve:

y = max/(1 + e−[(x−IC50)/b]) + min

where max is the maximal response observed, b is the slope of the curve and min the minimal response. GraphPad Prism 5.01 for Windows (GraphPad Software, San Diego, CA, USA) was used for all the statistical analyses and to calculate the I(E)C_20/50_ values.

## 3. Results

### 3.1. Characterization of Nanoparticles in the Stock Suspensions and Exposure Media

#### 3.1.1. Dynamic Light Scattering (DLS)

The hydrodynamic size measured by intensity (appearance >80%), the polydispersity index (PdI) and the Z-average of the NPs in the cell culture media are presented in Table 1. The PdI was very high for some of the NPs dispersed in the exposure media (TiO_2_-DDPA, CeO_2_-UNC and CeO_2_-DDPA), especially those containing toluene as a vehicle, which made it impossible to obtain a reliable size measurement. In general, no great differences were observed between the sizes of the NPs freshly prepared in cell medium (T0) and after 24 h incubation (T24). In general, the hydrodynamic sizes of the TiO_2_ NPs and CeO_2_ NPs were higher in the PLHC-1 medium. Independently of the medium used, the TiO_2_ and CeO_2_ NPs coated with CIT were smaller than the same core NPs uncoated, or coated with PEG. The three different Ag NPs showed very similar hydrodynamic sizes in both media (Table 1). In the *Daphnia magna* medium, TiO_2_-UNC, TiO_2_-PEG and CeO_2_-PEG produced big ensembles (Table 2). There were not big differences in size over time for any of the NPs, except for TiO_2_-DDPA and CeO_2_-DDPA, which aggregated after 48 h. It was not possible to obtain measurements of good quality for CeO_2_-UNC, or for NPs containing toluene in their formulation. Similarly, in the aquarium water (Table 3), it was not possible to obtain good quality data about the size of CeO_2_-UNC NPs. CIT coating stabilized the core Ag NPs over time. NPs coated with PEG were more polydispersed and unstable than those stabilized with CIT. All the NPs presented a negative ζ-potential in the different exposure media. Only the uncoated TiO_2_ and CeO_2_ NPs in *Daphnia magna* and fish media presented a positive charge (Table 4). Most of the NP suspensions were unstable, as shown by their ζ-potential < ±30 mV.

#### 3.1.2. TEM Analysis

**Figure 1:**
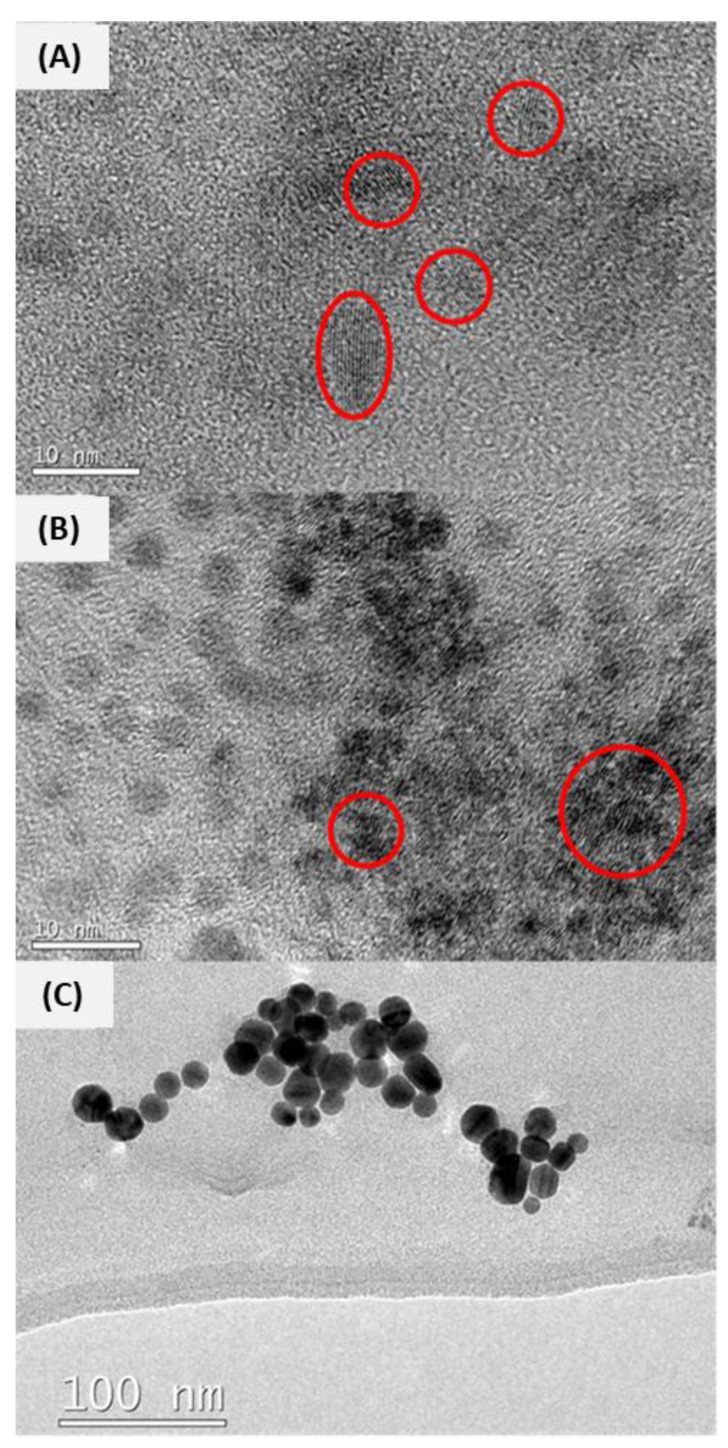
TEM images of the NPs tested in the stock suspension or in the exposure medium. (**A**) TiO_2_-UNC dispersed in the PLHC-1 cell medium at a concentration of 100 mg/L. Due to the extremely small size of the single NPs, the measurements of the NPs had to be conducted in base to their crystal layers (marked in red). The presence of the metal NPs was confirmed via EDX analysis. This comment is applied to all the TiO_2_ and CeO_2_ NPs. (**B**) Stock suspension of CeO_2_-CIT NP. (**C**) Stock suspension of Ag-CIT NP.

In the stock suspensions, all the TiO_2_ and CeO_2_ NPs presented extremely small sizes. They appeared entangled, but they could be identified individually by their crystal lattices, as can be observed, circled in red, in Figure 1A and 1B, respectively. The EDX analysis confirmed the presence of the respective metals. Sizes among uncoated and coated NPs with the same core were very similar, showing a mean diameter of 3.98 ± 0.20 nm for CeO_2_ NPs and 6.58 ± 0.85 nm for TiO_2_ NPs. All of them showed an amorphous shape. The shapes of Ag NPs were spherical (Figure 1C), with mean diameters of 20.9 ± 0.82 (*n* = 116) for Ag-CIT and 32.3 ± 3.59 (*n* = 63) for Ag-PEG. CeO_2_-DDPA and Ag-OAM NPs could not be identified in the stock suspensions because of the high levels of toluene in their matrix that broke the carbon grids. The sizes in the working concentrations prepared in the cell media for NPs (UNC: 6.12 ± 0.15, *n* = 90; CIT: 4.7 ± 0.62, *n* = 60; PEG: 3.5 ± 0.21, *n* = 50; DDPA: 3.4 ± 0.12, *n* = 107) and the CeO_2_-UNC NPs (2.9 ± 0.20, *n* = 91) ot significantly differ from the previously reported sizes in the stocks. However, the size of the CeO_2_-CIT NPs increased up to 111.5 ± 11.43 nm (*n* = 60). As well, CeO_2_-PEG NPs presented a mean size of 487.6 ± 198.6 nm. It should be noted that only big clusters (*n* = 3) could be observed in the images, and no single NPs were identified. Ag NPs dispersed in the cell exposure medium presented mean diameters of 24.7 ± 1.2 nm (*n* = 82) for Ag-CIT, 49.7 ± 2.7 nm (*n* = 106) for Ag-PEG and 24.1 ± 0.79 nm (*n* = 169) for Ag-OAM.

It was not possible to identify the NPs dispersed in the fish exposure medium. In the same way, only Ag-CIT (179.8 ± 94.18 nm, *n* = 77) and Ag-OAM (46.60 ± 15.30 nm, *n* = 82) were identified via TEM when they were dispersed in *Daphnia magna* medium. 

#### 3.1.3. Metal Analyses

The concentrations of NPs in the stock suspensions and in the exposure media were analyzed via ICP-OES or ICP-MS, respectively. All the nominal exposure concentrations (100 mg NP/L medium) were prepared based on the measured concentrations of the stock suspensions. This concentration was determined at time 0 and presented in Table 5. The triplicates of the exposure media of three independent cytotoxicity assays were collected and analyzed. The concentrations in both cell culture media were similar, and reproducible data were obtained among replicates (data only shown for the PLHC-1 cell line). For the cell lines and *Daphnia magna* assays, three measurements of the highest exposure concentration were taken at time 0, whereas for the fish, a daily measurement was carried out during the 96 h exposure period, in order to determine the extent to which the metals could be lost through precipitation or adsorption to the walls of the aquarium. There were no differences in concentration along the exposure time; therefore, a mean value was established, taking into account all the single results obtained (Table 5). For most of the NPs, the measured concentrations at time 0 were lower than expected. The loss of the TiO_2_ concentrations varied from 10% to 40%, whereas it was higher for CeO_2_ (10% to 78%). Very big losses in concentration were observed for the NPs coated with the hydrophobic coatings DDPA and OAM as well as for all Ag NPs, except Ag-CIT in the fish cell line medium.

### 3.2. Toxicity Assays

Due to the differences found between nominal and measured concentrations, data are expressed as mean ± SEM corrected to the measured concentrations at the highest concentration of NPs that the organisms were exposed to. Table 6 shows a summary of the hazard values obtained from the in vitro and in vivo assays (NOEC, I(E)C_20_ and I(E)C_50_).

#### 3.2.1. Cytotoxicity

NPs did not produce interferences with the cytotoxicity assays. 

**Figure 2:**
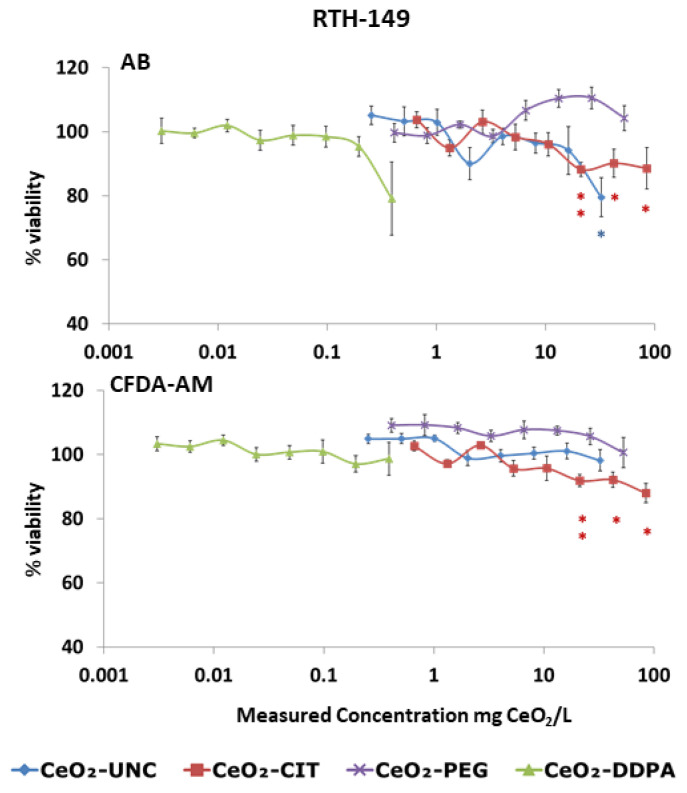
Cytotoxicity assays performed on RTH-149 cells exposed to CeO_2_ NPs and evaluated using the Alamar Blue (AB) and CFDA-AM assays. Measured concentrations are expressed as mg CeO_2_/L exposure medium. Average values of the % of viability with respect to the vehicle control of at least three independent experiments are presented (mean ± SEM). Asterisks denote statistical differences in comparison with the viability of cells exposed to the vehicle control. * *p* < 0.05, ** *p* < 0.01. The color of the asterisks relates them to the respectively colored NP.

TiO_2_ NPs did not produce toxicity in the two cell lines assayed; neither CeO_2_ NPs were toxic for the PLHC-1 cell line (Table 6). However, the RTH-149 cell line was more sensitive, presenting a significant decrease in viability after exposure to CeO_2_-UNC and CeO_2_-CIT (Figure 2, Table 6). CeO_2_-CIT NPs produced toxicity at two different cellular levels, affecting the mitochondria metabolic pathway (AB assay) and altering the cell membrane (CFDA-AM assay) with a NOEC_AB_, CFDA of 10.59 mg/L. However, CeO_2_-UNC NPs only affected the mitochondria activity (NOEC_AB_: 16.25 mg/L). The other two coated CeO_2_ NPs did not trigger any toxicity at the actual concentrations assayed. No effect was observed for any of the CeO_2_ NPs at the lysosomal level (NRU assay).

**Figure 3:**
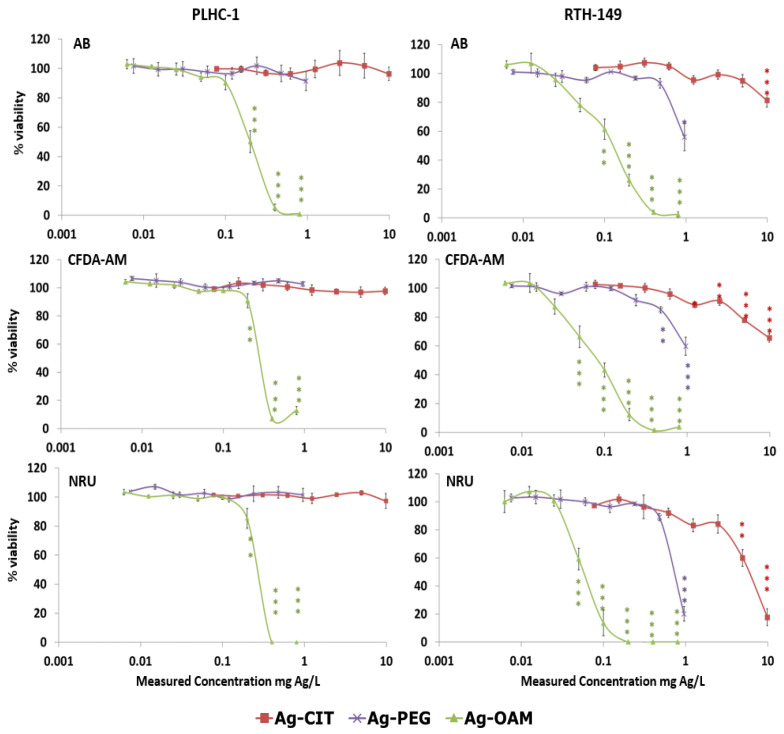
Cytotoxicity assays conducted in PLHC-1 and RTH-149 cells exposed to Ag NPs and evaluated using the Alamar Blue (AB), CFDA-AM and NRU assays. Measured concentrations are expressed as mg Ag/L exposure medium. The average values of the % of viability with respect to the vehicle control of at least three independent experiments are presented (mean ± SEM). Asterisks denote statistical differences in comparison with the viability of cells exposed to the vehicle control. * *p* < 0.05, ** *p* < 0.01, *** *p* < 0.001. The color of the asterisks relates them to the respectively colored NP.

A higher sensitivity of RTH-149 cells with respect to PLHC-1 cells was also visible after their exposure to Ag NPs (Figure 3, Table 6). In this case, only Ag-OAM provoked cytotoxicity in PLHC-1 cells. In contrast, the three Ag NPs led to decreases in viability in RTH-149 cells. Ag-CIT and Ag-PEG were more toxic at the membrane level, whereas the NOECs for Ag-AOM NPs were the same at the two membrane levels, plasmatic and lysosomal. The concentration of the vehicle with toluene, corresponding to the amount of solvent appearing in the highest concentration of Ag-OAM, resulted in toxicity. However, the consecutive dilutions of the vehicle were not toxic, indicating that the toxicity observed for the NPs at lower doses was due to the NPs themselves and not the solvent. 

#### 3.2.2. Acute Toxicity to Daphnia Magna

**Figure 4:**
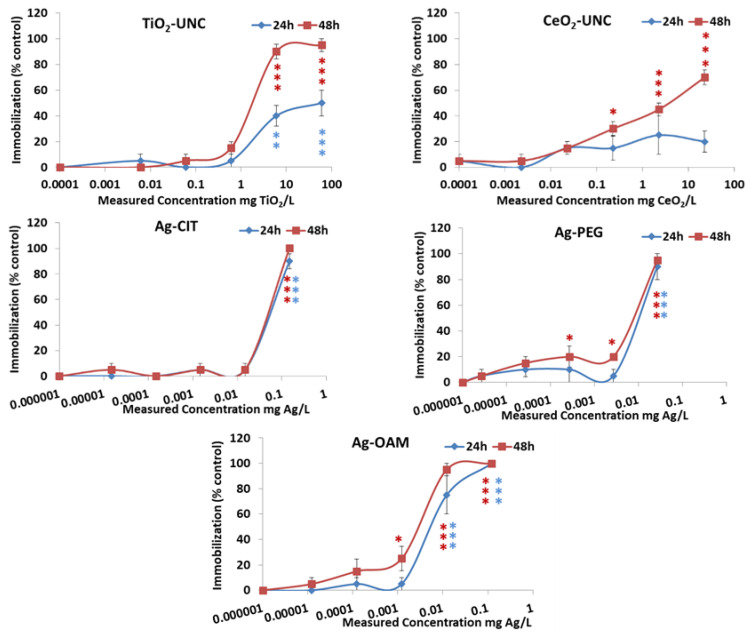
Data from *D. magna* immobilization assays after exposure to TiO_2_-UNC, CeO_2_-UNC and Ag NPs for 24 and 48 h. Measured concentrations are expressed as mg core composition/L exposure medium. The average values of four replicates are presented (mean ± SEM). * *p* < 0.05, ** *p* < 0.01, *** *p* < 0.001. The color of the asterisks relates them to the respectively colored curve.

Figure 4 and Table 6 show the results obtained from the *Daphnia magna* assays after acute exposure to the tested NPs. Coated metal oxide NPs showed no toxicity to the water flea at the maximum concentration tested, and effects on survival only appeared for the uncoated oxide NPs (Table 6). For TiO_2_-UNC and CeO_2_-UNC, the toxic effects increased highly over time; however, the toxicity of the Ag NPs was very similar after 24 h and 48 h of exposure (Figure 4). Ag NPs were, as evidenced also in the in vitro assays, the most toxic, following the pattern Ag-CIT < Ag-OAM < Ag-PEG according to NOECs. As for the cell lines, EC_50_ values confirmed an enhancement in toxicity by the PEG and OAM coatings with respect to the Ag-CIT NPs. The tested maximum concentration of the vehicle with toluene, corresponding to the highest concentration of Ag-OAM, resulted in toxicity; however, none of the following dilutions of the vehicle led to deaths.

#### 3.2.3. Acute Toxicity to Oncorhynchus Mykiss

Regarding the acute toxicity assays with rainbow trout, no toxicity at the limit dose (nominal concentration: 100 mg/L, measured concentrations reported in Table 5) was observed after exposure to the TiO_2_, CeO_2_ and Ag NPs (Table 6). TiO_2_ NPs and CeO_2_ NPs in the aquaria conferred a white and yellow color to the water, respectively. There was not an observed precipitation of particles during the experimental period, and nominal and measured concentrations were very close except for the UNC and PEG CeO_2_ NPs. Moreover, as indicated before, there was not a loss of concentration along the time of exposure. The assays with the Ag NPs coated with CIT or PEG did not produce any toxicity to the fish. The measured concentrations after 96 h of exposure were 0.035 mg/L of Ag-CIT and 0.08 mg/L of Ag-PEG, which are 40 and 3 times less concentrated than expected, respectively (Table 5). Water from the different tanks became black after only one day of exposure, probably due to the reaction of Ag ions with oxygen and water; however, conditions started to clear over time.

## 4. Discussion

It has been reported the need to correlate the shapes, sizes, compositions, and surface modifications of NMs with their environmental nanotoxicity [17]. The surface functionalization and the related changes in the physico-chemical properties might be very relevant for the toxicity of the NPs [18]. Thus, to provide a complete explanation of observed effects and mechanisms underlying the toxicity, NPs should be characterized in depth in their pristine form and in exposure media [6,19]. In general, there is still a lack of information regarding the behaviors of NMs in exposure media, and not so many studies report a complete characterization of the NMs once dispersed in exposure medium. 

In the present study, the results obtained via DLS, together with the TEM images of the NPs, confirmed the exposure to nano-scale materials. The NPs coated with citrate showed the smallest sizes of the aggregates, in comparison with those of uncoated and coated with PEG NPs. This was more apparent for TiO_2_ and CeO_2_ NPs in the freshly prepared dispersions, independently of the exposure media used. Ag-CIT ensembles were also smaller than the other Ag NPs when they were dispersed in cell medium; however, no differences were found in the sizes of the NPs populations when the dispersion was carried out in *Daphnia magna* and fish media. In relation to the dispersion stability, the magnitude of the ζ-potential has long served as an indicator against aggregation or deposition, with values above ±30 mV being considered moderately stable against aggregation due to charge stabilization. However, this was based on the behaviors of colloids of hundreds of nm in diameter, and are probably not appropriate for nanomaterials [20]. Table 4 shows that most of the NPs in the exposure media are not stable. The remark of Lowry et al. [20] can be confirmed with our results since values of ζ-potential >30 mV were not always related to a higher stability of the NP along the exposure time of the test. This was observed in the aquarium water and *Daphnia magna* medium, where NPs showing ζ-potential above ±30 mV were not stable during the exposure period. This is the case for CeO_2_-UNC, TiO_2_-CIT and CeO_2_-CIT in aquarium water (Table 3 and Table 4). TiO_2_-CIT and CeO_2_-UNC are other examples of discrepancies between stability of size and ζ-potential, in this case, in the *Daphnia magna* medium (Table 2 and Table 4). In contrast, the ζ-potential in cell medium was below ±30 mV for all the NPs (Table 4), but the size was stable during the 24 h exposure (Table 1). This fact can also be observed for other NPs in the *Daphnia magna* and fish media. 

Increased toxicity has been usually associated with decreasing particle size, although it is now recognized that this is not a rule. However, it seems that this is still a valid statement for Ag NPs. Indeed, data reported in the literature for the core Ag NPs under study suggest the following: the lower the size, the higher the toxicity. In this sense, Seitz et al. [21] reported a lower toxicity in *D. magna* (7.5 times less) for Ag NPs of 100 nm compared to Ag NPs of 20 nm. These results suggest that Ag NP toxicity could be grouped according to pristine size ranges. In fish, the different toxicity related to size has also been demonstrated with Ag NPs. Indeed, Kim et al. [22] exposed zebrafish embryos to Ag NPs differing in size and coating (20 nm-PVP, 20 nm-CIT, 110 nm-PVP, 110 nm-CIT). These authors demonstrated that the smaller 20 nm-Ag NPs were more toxic than the 110 nm-Ag NPs regardless of the surface coating. It is already known that some NMs, such as Ag, release ions to the medium once they are dispersed, and this can influence the toxicity of the material. In the present study, ion release was not measured, and we cannot conclude on the role of ions in the toxicity observed in cells and *Daphnia magna*. However, it has been reported that Ag NP solubility is influenced by particle diameter rather than coating or synthesis method [23]. These authors found an ion release after 24 h between 1 and 2% for Ag NPs of similar sizes (TEM) to those used in the present study. 

As pointed out, primary size by itself is not conclusive enough to confirm a potential similar behavior in terms of toxicity among NPs. Especially in the aquatic compartment, once the NPs reach this medium, they are very likely to form aggregates that will influence the toxicity. Therefore, the toxicity should be related not only to the size of the pristine NPs but also to the size of the NPs in the exposure medium [6]. This statement was reported by Lopes et al. [24] in a study with *Daphnia magna* that investigated the effect of ZnO NPs with two different particle sizes (30 nm and 80–100 nm), where NPs of different primary sizes but similar aggregated sizes showed the same EC_50_s. Also, Xiong et al. [25] found that the 96-h LC_50_ for zebrafish after exposure to ZnO NPs (30 nm primary size) or bulk ZnO (500 nm) were not significantly different, suggesting that the similar aggregation size found, once in suspension, could mitigate any potential difference. Nevertheless, this tendency of NPs with similar aggregation sizes to show the same toxicity was not confirmed when the experiment was repeated with TiO_2_ (NPs or bulk material), assuming the relevance of the specific core composition in the toxic effect [25]. Something similar can be concluded from the results obtained in the present study, where a general pattern of toxicity linked to the size of NPs in the different media could not be established for any of the tested NPs. Only in the case of CeO_2_-CIT, which formed smaller aggregates than the other three CeO_2_ NPs, a higher toxicity in RTH-149 cells could be observed. However, this tendency could not be confirmed neither in the PLHC-1 cells nor in *Daphnia magna* or fish.

In addition, the concentration of NPs in dispersion has been reported as important in their characterization and behavior. Fernandez-Cruz et al. [26] showed that it is essential to know measured concentrations to avoid making erroneous conclusions when only nominal concentrations are considered. This fact has also been observed in the present study. 

Another relevant NP physico-chemical characteristic for the toxicity exerted is the surface charge [27]. It has been reported that a higher NP toxicity is more associated to a positive ζ-potential compared to a negative one [28]. This association could not be confirmed with our results (Table 4 and Table 6). In fact, the positively charged NPs (uncoated TiO_2_ and CeO_2_) were toxic to *Daphnia magna*, whereas the CIT and PEG coated ones were not. This pattern was not observed in fish. 

Surface hydrophobicity is also an important parameter influencing toxicity, modifying the reactivity of the NPs with the surrounding medium and, therefore, changing the direct contact and effect with/on the target [27]. In our study, the hydrophobic Ag-OAM NPs were always much more toxic than the hydrophilic Ag-CIT and Ag-PEG NPs, probably due to an easier uptake of the NPs by the organisms. The results obtained with the hydrophobic TiO_2_/CeO_2_ NPs were not conclusive since the cells were exposed to very low concentrations. 

TiO_2_ NPs were not toxic to rainbow trout, even when the substance was visually available for the animals (water turned from transparent to a turbid white color). Toxicities were also not found in fish cell lines. The lack of acute toxicity after exposure to TiO_2_ NPs has been reported several times for different fish species, including rainbow trout [29,30]. Only Diniz et al. [31] has reported some mortalities in certain fish species. They found some mortality (<10%) in *Carassius auratus* (goldfish) exposed to 100 mg/L of TiO_2_ NPs, 53.3% mortality in *Danio rerio* (zebrafish) at this concentration and 10% at 10 mg/L. Different results were obtained in *D. magna*. TiO_2_-UNC NPs provoked the death of almost all the animals after exposure to the two highest concentrations. This was not the case for the coated NPs, allowing us to conclude that CIT and PEG have a protective effect against the TiO_2_ NPs’ toxicity in *Daphnia magna*. This could be probably explained in part by the negative charge conferred by these coatings.

Similar results were collected for CeO_2_ NPs. CeO_2_ NPs were not toxic to fish or PLHC-1, although, in this case, CeO_2_-UNC and CeO_2_-CIT NPs were toxic to RTH-149 and PEG, which provides again some protective face to the toxic effect. *Daphnia magna* responded to CeO_2_ NPs in a similar manner as TiO_2_ NPs. The uncoated nanoparticle was the only one presenting toxicity. Lee et al. [32] did not find acute toxicity in *D. magna* exposed to uncoated CeO_2_ NPs of other sizes; however, they reported mortality when exposed for a longer period (96 h) with 1 mg/L of NPs. Similarly, Van Hoecke et al. [33] found EC_50s_ of 40 mg/L and 71 mg/L in *D. magna* exposed for 21 d to CeO_2_ NPs of 14–20 and 29 nm, respectively. 

Regarding the Ag NPs tested, clear differences in toxicity were found depending on the fish species cell lines. Rainbow trout cells were more susceptible to Ag NPs than topminnow cells. PEG and OAM conferred a higher toxicity to the Ag NPs, while Ag-OAM were the only silver NPs exerting toxicity in PLHC-1 cells. The same effect was observed in *Daphnia magna*, with Ag-CIT being the less toxic NPs followed by Ag-PEG, and Ag-OAM being the most toxic ones. 

On the other hand, none of the tested Ag NPs were toxic to fish at the very low actual concentrations tested, lower than 0.08 mg/L (Table 5). The LC_50_ reported in rainbow trout juveniles is 2.16 mg/L for colloidal Ag NPs with alkylbenzene sulfonate (17 nm) [34]. Gagné et al. [35] also did not find mortality in rainbow trout exposed to 0.006 mg/L of Ag NPs. The decrease in the Ag NP concentration during the ecotoxicity studies has been related to the container materials and geometry/configurations and to the functionalized Ag NPs themselves, which influence their adsorption to the container [36]. This could explain the variability found between the nominal and measured concentrations in the present study, generally higher for the hydrophobic NMs and in vivo experiments, which use bigger containers. The exposure of fish to the different substances was visually confirmed for the three metal NPs. Aquaria with TiO_2_ NPs presented a milky color and turbidity, mostly in the case of the NPs coated with PEG. Media became yellow with the dispersion of CeO_2_ NPs. These colors did not significantly change during the experiment, showing the maintenance of NPs in dispersion during the whole exposure period. Aquaria with Ag NP suspensions also presented differences in color; however, in this case, there was a change over time, from dark to transparent. 

Summarizing the results of toxicity, TiO_2_-UNC NPs were only toxic to *Daphnia magna*, whereas the TiO_2_-CIT and TiO_2_-PEG NPs were not toxic to the four organisms. This finding allows for the conclusion that CIT and PEG coatings do not enhance the toxicity of the uncoated NPs, and, in the case of *Daphnia magna*, they protect against the core NP toxic effect seen. The DDPA coating influences highly in the formation of aggregates and loss of concentration. The actual concentrations tested were too low, lower than 0.28 mg/L. Therefore, no conclusions can be derived about the contribution of this coating to the toxicity observed for TiO_2_ NPs. Concerning CeO_2_ NPs, CeO_2_-UNC NPs were toxic to RTH-149 cell lines and *Daphnia magna* but not to PLHC-1 and fish, whereas CeO_2_-CIT and CeO_2_-PEG NPs were not toxic to the four organisms. The only exception was CeO_2_-CIT, which was toxic to the RTH-149 cells. As in the case of TiO_2_, CIT and PEG decreased the effect of the uncoated NPs. In addition, the actual concentrations tested for CeO_2_-DDPA NPs were too low to conclude on the effect of this coating in the toxicity of the uncoated NP. Finally, all Ag NPs were toxic to fish cell lines and *Daphnia magna*. In this case, the coatings PEG and OAM increased the toxicity of the Ag-CIT NPs. The hydrophobic OAM conferred the higher toxicity.

## 5. Conclusions

From the results, it may be concluded that the core NP composition is the main characteristic responsible for the toxicities observed. In this sense, Ag NPs were the most toxic followed by CeO_2_ and TiO_2_ NPs. 

With respect to the hydrophilic coatings, CIT and PEG, they do not increase the toxicity of the TiO_2_ and CeO_2_ uncoated NPs in the four aquatic organisms studied and, in the case of Daphnia magna, they eliminate the toxic effect. However, PEG increases the toxicity of Ag NPs in fish cell lines and Daphnia magna. With regard to the hydrophobic coatings DDPA and OAM, they favor the aggregation of NPs and loss of concentration. Ag-OAM NPs were more toxic than the hydrophilic PEG NPs. These results provide very useful information for safe design approaches. These approaches should consider that CIT and PEG are good candidates to decrease the toxicity of metal oxide NPs but not of metal NPs. This behavior should be further explored with other NMs.

Regarding species-specific effects, it was possible to identify a different susceptibility depending on the species exposed. Daphnia magna was more susceptible to TiO_2_-UNC and CeO_2_-UNC NPs than rainbow trout, but this was not the case for the coated NPs. An effort to verify the susceptibility of different aquatic species to different nanoforms should be made.

Finally, a relationship between in vitro/in vivo studies (fish cell lines vs. fish) could be established for the three TiO_2_ NPs but not for CeO_2_ NPs. It would be advisable to perform further studies to try to find an adequate fish cell line system to predict the toxicity of nanomaterials in fish.

## Figures and Tables

**Table 1:** Sizes (diameter, nm) of uncoated and coated NPs dispersed in the PLHC-1 and RTH-149 exposure cell media. Measures performed via DLS at the maximum exposure concentration directly after preparation (T0) and after incubation for 24 h (T24) at 30 °C (PLHC-1) or 20 °C (RTH-149) and 5% CO_2_. The average values of three independent measurements are presented (mean ± SEM). * PdI was close to 1, and therefore, the measured sizes are not confident enough.

		PLHC-1 Medium			RTH-149 Medium		
NM	Time (h)	PdI	Z-Average (nm; Mean ± SEM)	Main Peak Intensity (nm; Mean ± SEM)	PdI	Z-Average (nm; Mean ± SEM)	Main Peak Intensity (nm; Mean ± SEM)
TiO_2_-UNC	T0	0.25 ± 0.20	2409 ± 412	2159 ± 70	0.61 ± 0.03	603 ± 33	1047 ± 136
	T24	0.53 ± 0.10	2110 ± 400	1138 ± 70	0.68 ± 0.04	546 ± 49	958 ± 20
TiO_2_-CIT	T0	0.31 ± 0.01	281 ± 10	425 ± 22	0.25 ± 0.00	131 ± 7.6	171 ± 8
	T24	0.44 ± 0.02	719 ± 12	1324 ± 70	0.30 ± 0.05	178 ± 28	241 ± 48
TiO_2_-PEG	T0	0.16 ± 0.01	1190 ± 52	1227 ± 62	0.33 ± 0.04	941 ± 109	1215 ± 85
	T24	0.28 ± 0.01	1510 ± 16	1963 ± 50	0.34 ± 0.04	807 ± 47	1019 ± 93
TiO_2_-DDPA	T0	*	-	-	*	-	-
	T24	*	-	-	*	-	-
CeO_2_-UNC	T0	0.38 ± 0.03	2229 ± 234	1294 ± 219	*	-	-
	T24	0.34 ± 0.04	1658 ± 105	1117 ± 83	*	-	-
CeO_2_-CIT	T0	0.23 ± 0.02	596 ± 1	744 ± 21	0.47 ± 0.03	202 ± 23	354 ± 61
	T24	0.21 ± 0.02	590 ± 5	714 ± 12	0.43 ± 0.03	207 ± 25	343 ± 52
CeO_2_-PEG	T0	0.19 ± 0.05	821 ± 10	1015 ± 121	0.36 ± 0.02	431 ± 6	632 ± 22
	T24	0.11 ± 0.04	811 ± 23	870 ± 25	0.38 ± 0.05	452 ± 14	695 ± 59
CeO_2_-DDPA	T0	*	-	-	0.33 ± 0.08	242 ± 66	13 ± 3
	T24	*	-	-	0.67 ± 0.12	426 ± 11	11 ± 1
Ag-CIT	T0	0.32 ± 0.01	69 ± 1	99 ± 2	0.34 ± 0.01	61 ± 1	94 ± 2
	T24	0.31 ± 0.01	70 ± 1	104 ± 3	0.38 ± 0.05	64 ± 4	105 ± 16
Ag-PEG	T0	0.38 ± 0.01	325 ± 39	98 ± 13	0.51 ± 0.02	71 ± 5	118 ± 7
	T24	0.43 ± 0.01	421 ± 7	93 ± 30	0.29 ± 0.05	197 ± 55	108 ± 24
Ag-OAM	T0	0.33 ± 0.03	270 ± 21	187 ± 17	0.59 ± 0.10	413 ± 64	201 ± 87
	T24	0.38 ± 0.05	227 ± 45	589 ± 571	0.36 ± 0.01	198 ± 53	157 ± 5

**Table 2:** Sizes (diameter, nm) of uncoated and coated NPs dispersed in *Daphnia magna* exposure medium. Measures performed via DLS at the maximum exposure concentration, at time 0 (T0) and after 48 h of exposure (T48). The average values of three independent measurements are presented (mean ± SEM). * PdI was close to 1.

		*Daphnia magna* Medium		
NM	Time (h)	PdI	Z-Average (nm; Mean ± SEM)	Main Peak Intensity (nm; Mean ± SEM)
TiO_2_-UNC	T0	0.42 ± 0.10	7258 ± 640	3852 ± 591
	T48	0.46 ± 0.12	4681 ± 2711	1484 ± 871
TiO_2_-CIT	T0	0.16 ± 0.02	211 ± 101	255 ± 128
	T48	0.16 ± 0.01	322 ± 58	362 ± 67
TiO_2_-PEG	T0	0.29 ± 0.10	2914 ± 105	3115 ± 159
	T48	0.28 ± 0.06	4068 ± 512	3845 ± 427
TiO_2_-DDPA	T0	0.46 ± 0.18	441 ± 254	213 ± 47
	T48	*	-	-
CeO_2_-UNC	T0	*	-	-
	T48	*	-	-
CeO_2_-CIT	T0	0.20 ± 0.02	400 ± 32	462 ± 46
	T48	0.18 ± 0.02	482 ± 59	567 ± 84
CeO_2_-PEG	T0	0.53 ± 0.06	3939 ± 2467	1475 ± 448
	T48	0.21 ± 0.03	4451 ± 723	3915 ± 220
CeO_2_-DDPA	T0	0.60 ± 0.15	598 ± 252	256 ± 70
	T48	*	-	-
Ag-CIT	T0	0.47 ± 0.09	390 ± 128	222 ± 10
	T48	0.52 ± 0.02	512 ± 17	310 ± 7
Ag-PEG	T0	0.44 ± 0.01	345 ± 31	203 ± 19
	T48	0.62 ± 0.01	588 ± 24	224 ± 11
Ag-OAM	T0	*	-	-
	T48	*	-	-

**Table 3:** Sizes (diameter, nm) of uncoated and coated NPs dispersed in fish exposure medium. Measures performed via DLS at the maximum exposure concentration, at time 0 (T0) and every 24 h until the end of the experiment (T24, T48, T72, T96). The average values of three independent measurements are presented (mean ± SEM). * PdI was close to 1.

		*Oncorhynchus mykiss* Medium		
NM	Time (h)	PdI	Z-Average (nm; Mean ± SEM)	Main Peak Intensity (nm; Mean ± SEM)
TiO_2_-UNC	T0	0.68 ± 0.22	3095 ± 1650	1654 ± 1096
	T24	0.24 ± 0.08	4115 ± 281	3858 ± 36
	T48	*	-	-
	T72	0.46 ± 0.25	3286 ± 351	3246 ± 133
	T96	0.52 ± 0.27	4117 ± 1475	2331 ± 1502
TiO_2_-CIT	T0	0.25 ± 0.02	143 ± 18	12 ± 0.64
	T24	0.28 ± 0.01	27 ± 0.32	27 ± 0.55
	T48	0.26 ± 0.02	46 ± 9	59 ± 7
	T72	0.27 ± 0.01	266 ± 6	346 ± 8
	T96	0.49 ± 0.24	1942 ± 291	1969 ± 99
TiO_2_-PEG	T0	0.38 ± 0.23	2011 ± 877	1898 ± 1195
	T24	0.67 ± 0.10	933 ± 376	523 ± 245
	T48	0.33 ± 0.20	3948 ± 864	3702 ± 476
	T72	0.52 ± 0.20	2051 ± 691	1544 ± 1044
	T96	0.68 ± 0.24	2095 ± 16	769 ± 167
CeO_2_-UNC	All Ts	*	-	-
CeO_2_-CIT	T0	0.34 ± 0.02	76 ± 3	97 ± 1
	T24	0.25 ± 0.01	71 ± 0.11	87 ± 2
	T48	0.22 ± 0.01	141 ± 27	170 ± 32
	T72	0.34 ± 0.01	240 ± 19	377 ± 38
	T96	0.32 ± 0.05	1429 ± 295	1496 ± 575
CeO_2_-PEG	T0	0.41 ± 0.03	1501 ± 93	1084 ± 15
	T24	0.66 ± 0.03	1545 ± 21	886 ± 13
	T48	0.38 ± 0.22	1515 ± 1	758 ± 27
	T72/T96	*	-	-
Ag-CIT	T0	0.45 ± 0.01	360 ± 8	328 ± 29
	T24	0.37 ± 0.01	285 ± 2	260 ± 19
	T48	0.38 ± 0.03	273 ± 13	267 ± 20
	T72	0.37 ± 0.01	277 ± 3	286 ± 15
	T96	0.39 ± 0.01	289 ± 11	259 ± 21
Ag-PEG	T0	*	-	-
	T24	0.54 ± 0.01	370 ± 40	318 ± 111
	T48	*	-	-
	T72	0.59 ± 0.02	442 ± 11	167 ± 17
	T96	0.56 ± 0.02	389 ± 23	216 ± 21

**Table 4:** Zeta (ζ) potentials evaluated in the uncoated and coated (CIT and PEG) metal oxide NPs (TiO_2_ and CeO_2_) at the highest exposure concentration once dispersed in the exposure media. The results expressed as mV.

	Cell Medium (mV)		*Daphnia magna* Medium (mV)		*Oncorhynchus mykiss* Medium (mV)	
	Time 0	Time 24 h	Time 0	Time 48 h	Time 0	Time 96 h
Medium	−8.32		−10.35		−6.02	
TiO_2_-UNC	−8.96	−11.03	28.50	27.53	27.40	26.63
TiO_2_-CIT	−11.40	−14.87	−33.80	−35.13	−36.63	−28.2
TiO_2_-PEG	−13.37	−9.65	−24.60	−23.80	−24.23	−21.67
CeO_2_-UNC	−9.72	−10.13	41.30	40.93	34.70	37.43
CeO_2_-CIT	−10.32	−10.19	−21.27	−21.23	−33.13	−26.57
CeO_2_-PEG	−9.36	−9.455	−12.33	−12.1	−15.60	−20

**Table 5:** Nominal and measured levels at the maximum exposure concentrations, expressed as mg of the core (TiO_2_, CeO_2_ or Ag)/L exposure medium (mean ± SEM). Measured concentrations evaluated via ICP-OES.

Type of Assay		Nominal (mg/L)	Measured (mg/L)		Nominal (mg/L)	Measured (mg/L)		Nominal (mg/L)	Measured (mg/L)
Fish cell lines	TiO_2_-UNC	100	71.1 ± 18.9	CeO_2_-UNC	100	32.5 ± 2.9			
	TiO_2_-CIT	100	72.48 ± 9.3	CeO_2_-CIT	100	84.7 ± 5.2	Ag-CIT	10	9.9 ± 0.9
	TiO_2_-PEG	100	68.8 ± 0.03	CeO_2_-PEG	100	52.8 ± 2.7	Ag-PEG	1.62	0.96 ± 0.1
	TiO_2_-DDPA	100	0.28 ± 0.01	CeO_2_-DDPA	100	0.39 ± 0.16	Ag-OAM	8.6	0.80 ± 0.1
*D. magna*	TiO_2_-UNC	100	60.4 ± 8.5	CeO_2_-UNC	100	22.8 ± 9.5			
	TiO_2_-CIT	100	170.4 ± 5.5	CeO_2_-CIT	100	49.4 ± 1.9	Ag-CIT	1	0.15 ± 0.007
	TiO_2_-PEG	100	82.6 ± 9.8	CeO_2_-PEG	100	29.6 ± 3.4	Ag-PEG	0.16	0.03 ± 0.001
	TiO_2_-DDPA	100	0.17 ± 0.01	CeO_2_-DDPA	100	1.1 ± 0.12	Ag-OAM	0.86	0.12 ± 0.001
*O. mykiss*	TiO_2_-UNC	100	72.0 ± 6.1	CeO_2_-UNC	100	40.8 ± 3.4			
	TiO_2_-CIT	100	89.7 ± 6.6	CeO_2_-CIT	100	82.7 ± 8.8	Ag-CIT	1.5	0.035 ± 0.001
	TiO_2_-PEG	100	89.0 ± 11.8	CeO_2_-PEG	100	32.8 ± 3.3	Ag-PEG	0.24	0.08 ± 0.001

**Table 6:** NOEC, I(E)C_20_ and I(E)C_50_ values obtained in fish cell lines (PLHC-1 and RTH-149), *Daphnia magna* and *Oncorhynchus mykiss* exposed to uncoated and coated NPs. The results expressed as mg core NPs/L (mean ± SEM) corrected to the measured concentrations. The assays with fish cell lines and invertebrates were performed at least in triplicate.

	PLHC-1 (mg/L)	RTH-149 (mg/L)	*Daphnia magna* (mg/L)	*Oncorhynchus mykiss* (mg/L)
TiO_2_-UNC	NOEC > 71.1	NOEC > 71.1	NOEC = 0.60; EC_20_ = 1.7 ± 0.98; EC_50_ = 2.3 ± 1.09	NOEC > 72.0
TiO_2_-CIT	NOEC > 72.48	NOEC > 72.4	NOEC > 170.4	NOEC > 89.7
TiO_2_-PEG	NOEC > 68.8	NOEC > 68.8	NOEC > 82.6	NOEC > 89.0
TiO_2_-DDPA	NOEC > 0.28	NOEC > 0.28	NOEC > 0.17	Not tested
CeO_2_-UNC	NOEC ≥ 32.5	NOEC = 16.2; IC_20_ = 27.1 ± 4.8	NOEC = 0.023; EC_20_ = 0.72 ± 0.40; EC_50_ = 5.4 ± 1.8	NOEC > 40.8
CeO_2_-CIT	NOEC > 84.7	NOEC = 10.6	NOEC > 49.4	NOEC > 82.7
CeO_2_-PEG	NOEC > 52.8	NOEC > 52.8	NOEC > 29.6	NOEC > 32.8
CeO_2_-DDPA	NOEC > 0.39	NOEC > 0.39	NOEC > 1.1	Not tested
Ag-CIT	NOEC > 9.9	NOEC = 0.62; IC_20_ = 3.6 ± 0.31; IC_50_ = 6.1 ± 0.63	NOEC = 0.015; EC_20_ = 0.07 ± 0.02; EC_50_ = 0.08 ± 0.02	NOEC > 0.035
Ag-PEG	NOEC > 0.96	NOEC = 0.24; IC_20_ = 0.56 ± 0.03; IC_50_ = 0.73 ± 0.04	NOEC = 0.000027; EC_20_ = 0.006 ± 0.001; EC_50_ = 0.008 ± 0.002	NOEC > 0.08
Ag-OAM	NOEC = 0.10; IC_20_ = 0.16 ± 0.04; IC_50_ = 0.21 ± 0.01	NOEC = 0.025; IC_20_ = 0.034 ± 0.007; IC_50_ = 0.056 ± 0.005	NOEC = 0.00016; EC_20_ = 0.002 ± 0.0006; EC_50_ = 0.003 ± 0.0005	Not tested

## Data Availability

Data will be available upon request to the corresponding authors.

## Supplementary Materials

The following supporting information can be downloaded at https://www.mdpi.com/article/10.3390/toxics12020142/s1, Table S1: Labeled information about nanoparticles, vehicles and concentrated coatings supplied by PlasmaChem GmbH (Berlin, Germany).
