# Supplementary material for: Toxic Effects of Different Coating-Related Functionalized Nanoparticles on Aquatic Organisms
Source: Toxics. 2024 Feb 9;12(2):142. doi: 10.3390/toxics12020142 (PMC10891610; doi:10.3390/toxics12020142)
Supplement: Supplementary file 1 [file toxics-12-00142-s001.zip › toxics-2826957-supplementary.pdf]

Table S1. Labeled information about nanoparticles, vehicles and concentrated coatings supplied by PlasmaChem GmbH (Berlin, Germany)

	**NPs PlasmaChem**	**Vehicle**	**Concentrated Vehicle (Full coating concentration)**
**TiO_2-UNC**	TiO_2 (anatase, ca. 4-8 nm, uncoated) colloid solution in water media 200 mg/mL, pH≤ 1.0	Nitric acid 6.3 mg/mL solution	Concentrated vehicle corresponding to TiO_2 particles, uncoated; nitric acid solution 30.2 mg/mL
**TiO_2-CIT**	TiO_2 (anatase, ca. 4-8 nm, modified by citrate) colloid solution in water media ca. 145 mg/mL, pH≈ 7-8	Na citrate solution in water 2.3 mg/mL	Concentrated vehicle corresponding to TiO_2 particles, modified by citrate; Na citrate solution in water 84.8 mg/mL
**TiO_2-PEG**	TiO_2 (anatase, ca. 4-8 nm, modified by mPEG phosphoric acid ester) colloid solution in water media 100 mg/mL, pH≈ 7-8	mPEG phosphoric acid ester in water 0.025 mg/mL	Concentrated vehicle corresponding to TiO_2 particles, modified by mono mPEG phosphoric acid ester, 51.6 mg/mL
**TiO_2-DDPA**	Hydrophobic TiO_2 (anatase, ca. 4-8 nm, modified by dodecylphosphonic acid) colloid solution in toluene ca. 200 mg/mL	dodecylphosphonic acid in toluene 0.5 %	Concentrated vehicle corresponding to hydrophobic TiO_2 particles, modified by dodecylphosphonic acid in toluene, 89.0 mg/mL
**CeO_2-UNC**	CeO_2 (anatase, ca. 4-8 nm, uncoated) colloid solution in water media 100 mg/mL w/v, pH≈ 1-2	Nitric acid 6.3 mg/mL solution	Concentrated vehicle corresponding to CeO_2 particles, uncoated; nitric acid solution 16.8 mg/mL
**CeO_2-CIT**	CeO_2 (anatase, ca. 4-8 nm, modified by citrate) colloid solution in water media ca. 50 mg/mL, pH≈ 7-8	Na citrate solution in water 0.8 mg/mL	Concentrated vehicle corresponding to CeO_2 particles, modified by citrate; Na citrate solution in water 22.9 mg/mL
**CeO_2-PEG**	CeO_2 (anatase, ca. 4-8 nm, modified by mPEG phosphoric acid ester) colloid solution in water media 50 mg/mL, pH≈ 7-8	mPEG phosphoric acid ester in water 0.012 mg/mL	Concentrated vehicle corresponding to CeO_2 particles, modified by mono mPEG phosphoric acid ester, 19.8 mg/mL
**CeO_2-DDPA**	Hydrophobic CeO_2 (anatase, ca. 4-8 nm, modified by dodecylphosphonic acid) colloid solution in toluene ca. 200 mg/mL	dodecylphosphonic acid in toluene 0.5 %	Concentrated vehicle corresponding to hydrophobic CeO_2 particles, modified by dodecylphosphonic acid in toluene, 81.8 mg/mL
**Ag-CIT**	Ag particles (ca. 10 nm) stabilized by citrate in water media ca. 0.1 mg/mL	Na citrate solution in water 0.56 mg/mL	Concentrated vehicle corresponding to Ag particles stabilized by citrate: Na citrate solution in water 0.56 mg/mL
**Ag-PEG**	Ag particles (ca. 10 nm) stabilized by PEG-mercaptopropionic ester in water media ca. 0.2 mg/mL	PEG-mercaptopropionic ester in water 0.005 mg/mL	Concentrated vehicle corresponding to Ag particles stabilised by mPEG-mercaptopropionic ester: PEG-mercaptopropionic ester in water 0.088 mg/mL
**Ag-OAM**	Hydrophobic Ag particles (ca. 10 nm) stabilized by oleylamine in toluene ca. 1 mg/mL	Oleylamine in toluene 0.025 mg/mL	Not available
